# Rapid Acquisition and Identification of Structural Defects of Metro Tunnel

**DOI:** 10.3390/s19194278

**Published:** 2019-10-02

**Authors:** Qing Ai, Yong Yuan

**Affiliations:** 1 Department of Civil Engineering, School of Naval Architecture, Ocean and Civil Engineering, Shanghai Jiao Tong University, Shanghai 200240, China; ai.qing@sjtu.edu.cn; 2 College of Civil Engineering, Tongji University, Shanghai 200092, China; 3 State Key Laboratory of Disaster Reduction in Civil Engineering, Tongji University, Shanghai 200092, China

**Keywords:** inspection method, photogrammetry, image processing, metro tunnel, structural defects

## Abstract

Metro systems in urban cities demand rapid inspection methods, in order to identify critical structural defects in a timely manner. However, traditional inspection methods are only specific to one kind of structural defect, which reduces the overall efficiency of inspection. This study proposes an integrated solution for rapidly acquiring and identifying two kinds of structural defects (surface defects and cross-sectional deformation) in a metro tunnel, using a cart equipped with non-metric cameras. The integrity and rapidity are considered in formulating a systematic design for the development of the acquisition device. Methodologies based on image processing and photogrammetry are proposed to identify the structural defects of the metro tunnel. A series of on-site tests validate that the proposed method has enough speed and has acceptable accuracy in detecting critical structural defects of metro tunnels. The cost and efficiency analysis shows that the proposed method is competitive, which will greatly improve the efficiency and reduce the costs of the inspection of metro tunnels.

## 1. Introduction

Metro systems are an important type of infrastructure in large cities. Accidents caused by untimely inspection have recently attracted the attention of researchers, owners and administrations [1,2,3,4,5]. It has been widely recognized that the structural defects of metro structures (mainly tunnels) should be frequently inspected, in order to avoid the risk of disruption due to late intervention [6,7,8,9,10,11,12,13]. However, the time required for inspection leads to the unavailability of transport infrastructures. The inspection of metro tunnels is tense and urgent, as the time period of inspection is restricted to only several hours, due to daily operating pressures. For example, the time period available for inspecting structural defects is only 2 h for the Shanghai Metro, which has an operating mileage of 705 km (as of 2018). Considering the fact that manual inspection is still dominant in the current practice [14], rapid inspection devices and methodologies should be developed to meet the requirements of the safe and efficient operations of such critical infrastructure.

Typical structural defects of a metro tunnel are surface defects and deformations. The former includes leakages, cracks and spallings on the surface of concrete structures [15,16,17]. The latter includes cross-sectional deformation, uneven settlement and stagger between adjacent structural sections [18,19,20]. Different kinds of devices and methods for identifying specific structural defects have been developed in recent years [21,22,23,24]. For example, surface defects in a metro tunnel can be identified by optical imaging, infrared imaging and laser imaging-based devices or methodologies [25,26,27,28,29,30,31,32,33,34,35,36,37,38]. Many relatively mature image processing-based algorithms have been applied for detecting defects, such as leakages and cracks. In contrast, there have been few rapid methods developed for identifying the deformations of metro tunnels [39,40,41,42,43,44,45,46,47]. Currently, measuring the deformation of a metro tunnel is still carried out by traditional methods, such as total station, which is time-consuming in typical current situations. Some commercial inspection devices, which adopt a rotating laser scanner as a sensor, are available [48,49,50,51]. These devices are able to acquire high-quality raw image data of a metro tunnel but are limited by slow scanning speeds and a lack of efficient processing algorithms [21]. Thus, both their acquisition speeds and identification methods cannot satisfy the current demand. Additionally, those inspection devices and methods only deal with one specific type of structural defect. Considering the limited time available, the inspection of metro tunnel demands integrated devices and methods, in order to comprehensively inspect several structural defects through one inspection.

To overcome the shortcomings of the existing devices and methods, this study proposes an integrated device for rapid acquisition and identification of two kinds of structural defects (surface defects and cross-sectional deformation) of metro tunnels. The device is designed by a systematic consideration of integrity and rapidity to improve the speed of data acquisition. After acquisition, the surface defects are identified by algorithms based on the image differencing strategy and cross-sectional deformations are calculated by transmissive projection using calibrated non-metric cameras. The functionality and availability of the proposed method are validated through on-site applications. Finally, the cost, speed and accuracy of proposed method are compared with traditional methods, in order to assess its advantages.

## 2. Solution for Rapid Acquisition

The integrated device adopts a railway-compatible removable cart to acquire information of the metro tunnel. As it is designed to acquire two different kinds of structural defects in a metro tunnel, it is necessary to comprehensively consider the spatial arrangement of the components, functional co-ordination and other issues of the device.

### 2.1. Integrity

**Figure 1:**
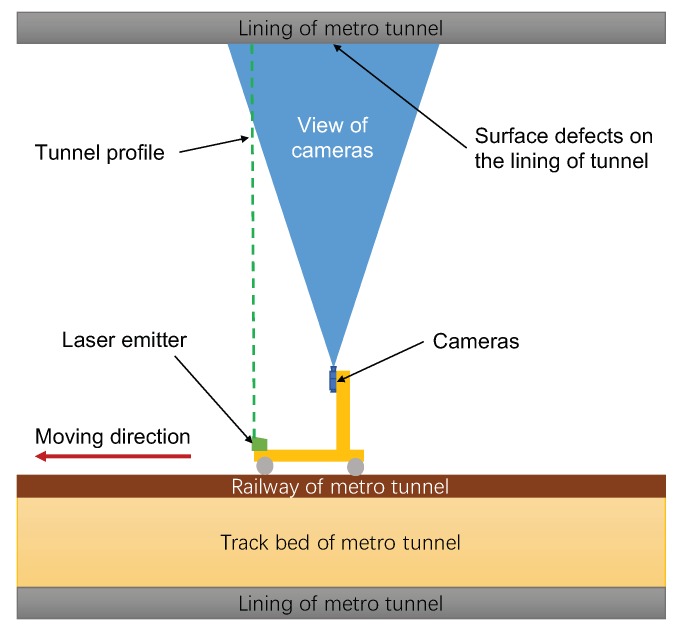
The design of obtaining surface defects and the cross-sectional profile, from the aspect of integrity.

Non-metric charge-coupled device (CCD) cameras are employed as the imaging sensors; however, there are conflicts in the spatial arrangement for acquiring surface defects and the cross-sectional profile of the metro tunnel. The acquisition range of surface defects is a circular belt on the inner surface of the metro tunnel and the common scheme is to use several cameras in a circular arrangement, with their axes perpendicular to the longitudinal axis of the tunnel. However, acquiring a cross-sectional profile of a metro tunnel requires an appropriate angle (about 45${}^{\circ }$) between the tunnel profile and the axis of the camera, which means that the laser emitter (generating the tunnel profile) must be placed at an appropriate distance from the cameras. Therefore, the laser emitter has to be arranged at the front of the integrated device and the images of surfaces defects and the cross-sectional profile can both be obtained in view of the cameras, as shown in Figure 1.

**Figure 2:**
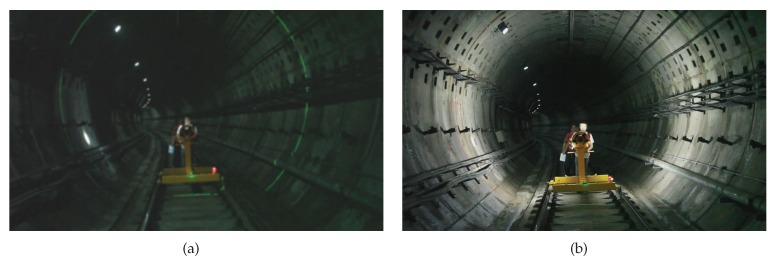
Integrated acquisition during inspection: (**a**) Mode of acquiring surface images and (**b**) mode of acquiring profile images.

To collaboratively achieve the function of acquiring two defects during one inspection, the integrated device is designed to have two modes: acquiring surface images and acquiring profile images. In the mode of acquiring surface images, the illumination components will generate a uniform lighting environment on the surface of metro tunnel to capture high-quality images of defects. In the mode of acquiring profile images, the illumination components will stop working and the laser emitter will generate a bright profile on the wall of the tunnel, from which the cross-sectional profile of the tunnel can be easily extracted. The integrated device adopts an alternate strategy to switch the mode of acquisition simultaneously by calculating the mileage; thus, the two kinds of structural defects can be acquired during one inspection, as shown in Figure 2.

Accurately calculating the mileage traveled by the integrated device is the key to control the modes of acquisition. Thus, the mileage within the tunnel is captured by a high-resolution encoder, attached to the wheel of the cart. When the integrated device moves on the railway, a pulse number *N* is sent out by the encoder, which is counted and transferred into a mileage *S*. The conversion relation between the pulse number and mileage can be calculated using the wheel diameter *D*, as shown in Equation (1). Assuming an encoder with a resolution of $R_{p}=4096$ line/rad attached to a wheel with diameter of 150 mm, the accuracy of the positioning is 0.115 mm/pulse.

(1) $$S=\frac{\pi D}{R_{p}}\cdot N.$$

### 2.2. Rapidity

Generally, the technical measures for improving the rapidity of the integrated device are:

- Acquiring data in a moving process, rather than setting up stations;
- Rapid speed of imaging by the cameras equipped on the integrated device; and
- Algorithm-based processing, rather than manual work.

For further analysis, the total time of inspection can be divided into two parts: working time (on-site) and processing time (in office). The rapidity of the integrated device is achieved by reducing the time cost in these two aspects. Due to the limited inspection time on-site, reducing the working time on-site is more important. Therefore, the speed of acquiring images should be increased and the on-site preparation time should be reduced, as much as possible.

The speed of acquiring images depends on many factors, such as the frame-rate of the camera, the transmission speed and the write speed of the hard disk. For example, during the moving process, high speed of the integrated device requires a high frame-rate of the camera, to ensure full coverage during acquisition. Assuming that the computing power is fully invested in the data acquisition and storage during inspection, the speed of acquiring images can be ideally estimated by the following equation:

(2) $$S_{sys}=min\left\{S_{cam},S_{trans},S_{wr}\right\},$$

where $S_{sys}$ is the speed of acquiring images by the integrated device, $S_{cam}$ is the frame-rate of the camera, $S_{trans}$ is the transmission speed and $S_{wr}$ is the write speed of the hard disk. The speed of acquiring images is, thus, restricted to the above three aspects and deficiency in one of these aspects will result in a decrease of speed. In contrast, to improve the speed of acquiring images, the three aspects should be comprehensively improved: a high frame-rate camera is better than low frame-rate one, a faster transmission method is better than a lower one and a SSD (Solid State Disk) with higher write speed is better than a HDD (Hard Disk Drive).

Reducing the preparation time on-site is also meaningful. The preparation time is mostly spent on the assembly and disassembly of the device. Therefore, the device is designed with several separate parts, which can be connected quickly by fast chutes and plugs. The on-site application validated that the assembly and disassembly of the integrated device took a total of 20 min, which left more time for acquisition.

In addition, algorithm-based processing of the data is obviously more efficient than manual work. This will reduce the processing time in office and also reduce manual errors, which may be caused by insufficient professional skills in some cases.

## 3. Methods of Identifying Structural Defects

### 3.1. Surface Defects of Concrete Linings

#### 3.1.1. Image Differencing Strategy

**Figure 3:**
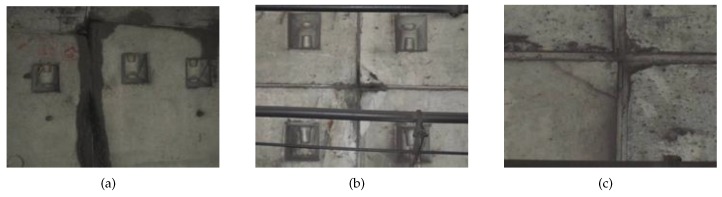
Surface defects in a metro tunnel: (**a**) Leakage, (**b**) Spalling and (**c**) Crack.

The surface defects in a metro tunnel are leakages, spallings and cracks, among others, which are shown in Figure 3. They can be identified by a series of image processing operations based on the image differencing strategy.

**Figure 4:**
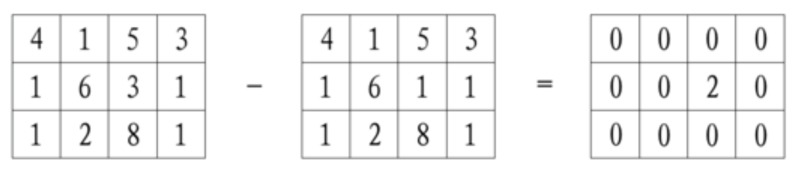
Image differencing strategy.

The image differencing strategy has been widely used to detect differences between two images with identical backgrounds. It generates a new image by subtracting the gray values of the two images. The principle is shown in Figure 4, where the numbers in the squares represent the gray values of pixels in the image; the gray values of pixels in corresponding positions are subtracted. If the differences of the gray values are not zero, the positions of changes (for example, leakages) can be identified in the image.

In practice, an image of tunnel lining without defect is chosen as the background. Then, images acquired by an integrated device are compared with this background to generate new images for further processing. The implementation of the image differencing strategy also benefits from the position subsystem, which helps to take images at the same location. As surface defects gradually appear, the image differencing strategy is an efficient method for identifying the changes (i.e., emerging defects) in a metro tunnel.

#### 3.1.2. Image Preprocessing

The first step of image processing is gray processing, which is the basis for subsequent image processing. This operation converts the colored images into gray images, for convenience of computation. Generally, it is described by Equation (3):

(3) Y=0.299R+0.587G+0.114B,

where *Y* is the gray value of a colored pixel and *R*, *G* and *B* are three primary components that a colored pixel consists of.

#### 3.1.3. Image Segmentation

With the aid of positioning by the encoder, images were acquired at regular places in a metro tunnel. To avoid the interference of joints and bolt holes in the metro tunnel, the background and target images should be aligned before implementing the image differencing strategy. We used a difference minimization algorithm to find the best alignment location of the two images. In this method, the image of background $B_{i}$ is moved over the image of target $I_{i}$, within a restricted area, to search the best location; the objective function *Q* is the total difference of every pixel value of the two images. When the objective function is minimized, then we believe that the two images are aligned, as shown in Equation (4):

(4) $$min\{Q=f\left(I_{i},B_{i}\right)\}.$$

The image differencing strategy is conducted after gray processing and alignment and the result is a new image generated by the differential values. A threshold θ is introduced to detect the defects, assuming that obvious changes are mainly caused by the appearance of surface defects. After the thresholding operation of Equation (5), the new image $C_{i}$ becomes a binary image:

(5) $$C_{i}=\left\{\right|I_{i}-B_{i}\left|⩾\theta \right\}.$$

To distinguish the defects in the binary image, these defects need to be labeled. Therefore, the concept of a connected region is proposed. A connected region is a collection of connected pixels, separated from the others. The algorithm of labeling a connected region is presented in the following steps:

(1) Each pixel of an image is expressed as $P_{i,j}$.

(2) For (i=0,j=0), if $P_{i,j}=1$ and $P_{i,j}$ has not been labeled before, this pixel $P_{i,j}$ is labeled as connected region *N* (*N* starts from 1).

(3) Search the eight neighboring pixels of $P_{i,j}$ (i.e., $P_{i-1,j-1}$, $P_{i-1,j}$, $P_{i-1,j+1}$, $P_{i,j-1}$, $P_{i,j+1}$, $P_{i+1,j-1}$, $P_{i+1,j}$ and $P_{i+1,j+1}$). If any one of the eight neighboring pixels is 1, this neighboring pixel is labeled as connected region *N*.

(4) If the neighboring pixel value in process (3) is 1, then search its eight neighboring pixels and repeat process (3) until no neighboring pixel is 1. The total number of pixels labeled as connected region *N* is counted as $C_{N}$.

(5) Repeat steps (2), (3) and (4). The new connected region is labeled as N+1; if there is no new connected region, return 0.

(6) Stop when process (5) returns 0.

**Figure 5:**
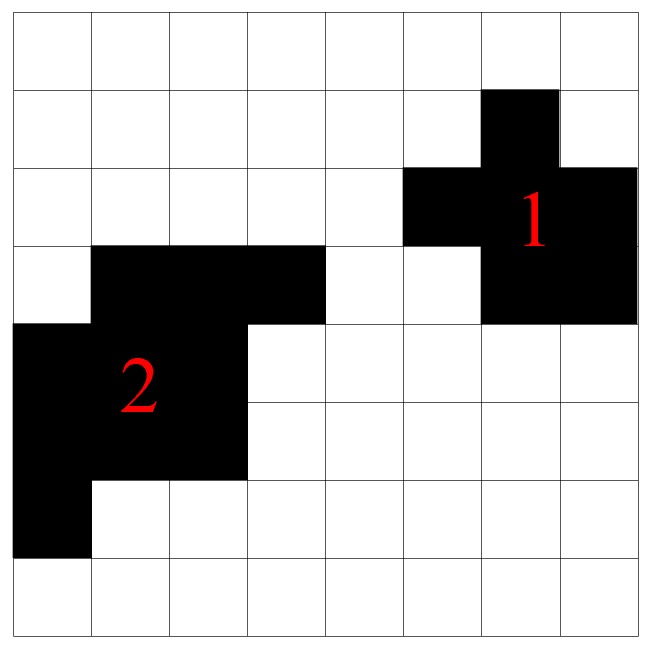
Connected regions in an image.

Figure 5 shows an image containing two connected regions: connected region 1 and connected region 2.

It should be noted that the alignment is not effective for irregularly arranged pipes or other facilities in a metro tunnel, which introduce false positives after implementing the image differencing strategy. Thus, after labeling connected regions, some small connected regions should be deleted, as they can be considered as false alarms caused by these interferences in the detection of surface defects.

#### 3.1.4. Classification of Surface Defects

The features of a connected region can be calculated, and the extracted features are then used to classify the surface defects, as listed in Table 1. The area is defined as the total number of pixels of the connected region. The lengths of the long and short axes are defined as the length and width of the smallest rectangle enveloping the connected region, respectively. The fill rate is defined as the ratio of $C_{N}$ to the number of pixels in the smallest rectangle enveloping the connected region. Long axis/Short axis is the ratio of the lengths of the long and short axes.

It should be noted that the features of different type of defects in Table 1 are summarized from manual experience and are not critical rules for identifying defects. In addition, according to the actual size of the image, the areas of surface defects can be converted into engineering units for further applications.

### 3.2. Cross-Sectional Profile of Metro Tunnel

#### 3.2.1. Basic Principles of Transmissive Projection

**Figure 6:**
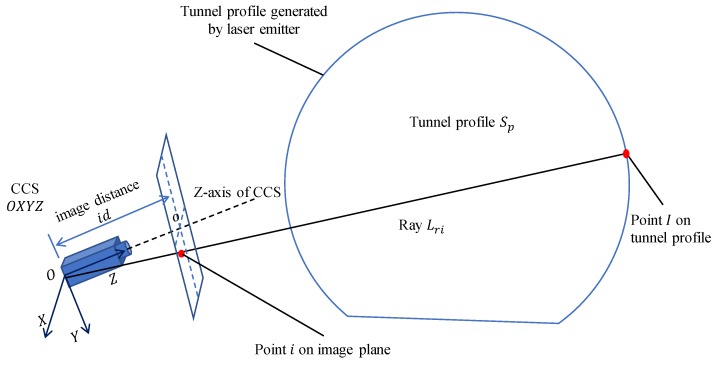
Transmissive projection of tunnel profile.

This study adopts transmissive projection in photogrammetry to identify the cross-sectional profile of the metro tunnel. In photogrammetry, assuming we have a calibrated camera and a fixed-position laser emitter, the laser emitter generates a laser plane, $S_{p}$, which intersects a bright tunnel profile on the inner surface of the metro tunnel. Given the schematic of transmissive projection in Figure 6, the point *I* on the tunnel profile can be calculated by Equation (6):

(6) $$\left\{\begin{array}{c} L_{ri}:\frac{x}{A_{i}}=\frac{y}{B_{i}}=\frac{z}{C_{i}}\\ S_{p}:H\cdot x+I\cdot y+J\cdot z+K=0 \end{array}\right.,$$

where $L_{ri}$ is the ray from the origin *O* of the camera co-ordinate system (CCS) to point *I*, which can be calculated by the image point *i* on the image plane. It is assumed that the parameters of the image plane and the equation of tunnel profile $S_{p}$ are already known, based on the following calibrating processes.

#### 3.2.2. Calibration of Imaging Subsystem

(1) Calibration of camera parameters.

To let the non-metric camera have the function of measurement, the image distance id and image distortion array $X_{\mathrm{dist}}=\left[\left(x,y\right)\left(u,v\right)\right]$ should be calibrated.

The image distance id is used to estimate the equation of the ray $L_{ri}$. In Figure 6, assuming that the co-ordinate of point *i* on the image plane is (m,n), its co-ordinate in the CCS OXYZ should be (m,n,id). Thus, the equation of ray $L_{ri}$ can be expressed by:

(7) $$\frac{x}{m}=\frac{y}{n}=\frac{z}{id}.$$

**Figure 7:**
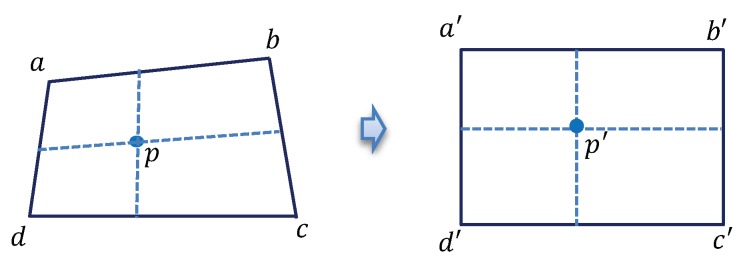
Bilinear interpolation method for calibrating image distortion.

Non-metric cameras usually have serious image distortion, which is an effect of bending light near the edges of the lens; however, we encounter it near the center of the lens. It can be calibrated using the bilinear interpolation method. Figure 7 explains the bilinear interpolation method by two meshes, representing the distorted image (left, quadrangle one) and the calibrated image (right, rectangle one). The vertices a,b,c,d and $a^{\prime },b^{\prime },c^{\prime },d^{\prime }$ are the corresponding control points of the two meshes. Assuming *p* is a pixel in the quadrangle abcd, after calibrating the image distortion, it is transferred to its corresponding point $p^{\prime }$ in the rectangle $a^{\prime }b^{\prime }c^{\prime }d^{\prime }$ by bilinear interpolation. This operation is conducted on every pixel of the distorted image and the image distortion array $X_{\mathrm{dist}}=\left[\left(x,y\right)\left(u,v\right)\right]$ is the mapping of every *p* and $p^{\prime }$.

(2) Calibration of transformation matrix between cameras.

**Figure 8:**
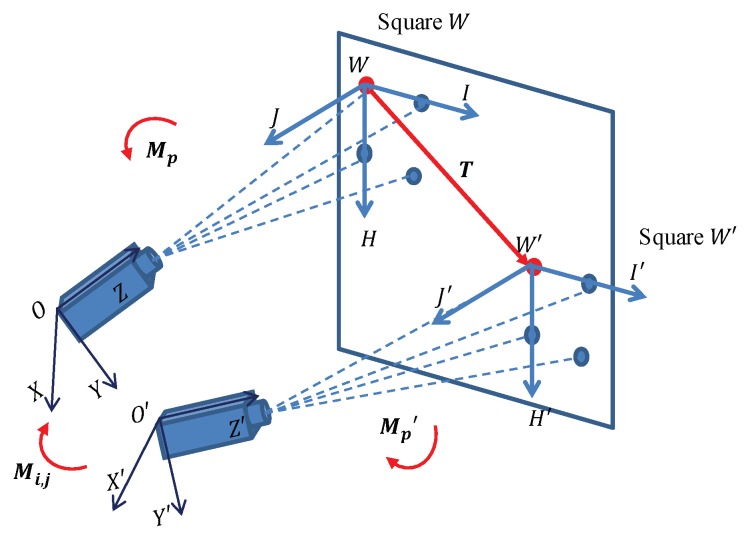
Calibration of transformation matrix between cameras.

As each camera has its own camera co-ordinate system (CCS), transformations between different CCS require calibration, which is the basic requirement for presenting the results in a unified co-ordinate system. A special target, containing eight points on a plane, was designed for calibration, as shown in Figure 8. Every four points on the target constitute a square and form a target co-ordinate system (TCS). The geometric information of the two squares is known before calibration. During calibration, two cameras separately take images of the two squares and the images are analyzed to give the transformation matrix based on the following method.

As shown in Figure 8, there are four co-ordinate systems, which are CCS OXYZ, CCS $O^{\prime }X^{\prime }Y^{\prime }Z^{\prime }$, TCS HIJK and TCS $H^{\prime }I^{\prime }J^{\prime }K^{\prime }$. Assume that $M_{p}$ is the transformation matrix from TCS WHIJ to CCS OXYZ and, similarly, that $M_{p}^{\prime }$ is the transformation matrix from TCS $W^{\prime }H^{\prime }I^{\prime }J^{\prime }$ to CCS $O^{\prime }X^{\prime }Y^{\prime }Z^{\prime }$. As the parameters of the cameras have been calibrated and the geometric information of the targets are already known, the co-ordinates of the four vertices of the square *W* in CCS OXYZ and four vertices of the square $W^{\prime }$ in CCS $O^{\prime }X^{\prime }Y^{\prime }Z^{\prime }$ can be computed by transmissive projection. Using the co-ordinates of the four vertices in CCS, the transformation matrix from TCS to CCS can be computed, which consists of a rotation vector R and a translation vector t, as shown in Equation (8).

(8) $$\left\{\begin{array}{c} R=\left[\begin{array}{ccc} u_{\mathrm{xx}} & u_{\mathrm{xy}} & u_{\mathrm{xz}}\\ u_{\mathrm{xy}} & u_{\mathrm{yy}} & u_{\mathrm{yz}}\\ u_{\mathrm{xz}} & u_{\mathrm{yz}} & u_{\mathrm{zz}} \end{array}\right]\\ t=(t_{x}\;t_{y}\;t_{z}) \end{array}\right..$$

Based on Equation (8), we assume that the computed transformation matrix from TCS WHIJ to CCS OXYZ is $M_{p}=\left(R_{p},t_{p}\right)$ and the computed transformation matrix from TCS $W^{\prime }H^{\prime }I^{\prime }J^{\prime }$ to CCS $O^{\prime }X^{\prime }Y^{\prime }Z^{\prime }$ is $M_{p}^{\prime }=\left(R_{p}^{\prime },t_{p}^{\prime }\right)$. The translation vector from TCS WHIJ to TCS $W^{\prime }H^{\prime }I^{\prime }J^{\prime }$ is T, which is already known, as the squares *W* and $W^{\prime }$ were designed to be parallel. Thus, the transformation matrix $M_{i,j}$ from CCS $O^{\prime }X^{\prime }Y^{\prime }Z^{\prime }$ to CCS OXYZ can be computed by vector computation, as shown in in Equation (9):

(9) $$\left\{\begin{array}{c} R_{i,j}={\left(R_{p}^{\prime }\right)}^{-1}\times R_{p}\\ t_{i,j}=t_{p}+T\times R_{p}-t_{p}^{\prime }\times {\left(R_{p}^{\prime }\right)}^{-1}\times R_{p} \end{array}\right..$$

Then, the point *q* in CCS $O^{\prime }X^{\prime }Y^{\prime }Z^{\prime }$ can be transformed as a point *p* in CCS OXYZ by:

(10) $$p=q\times R_{i,j}+t_{i,j}.$$

(3) Calibration of relations between camera and tunnel profile.

**Figure 9:**
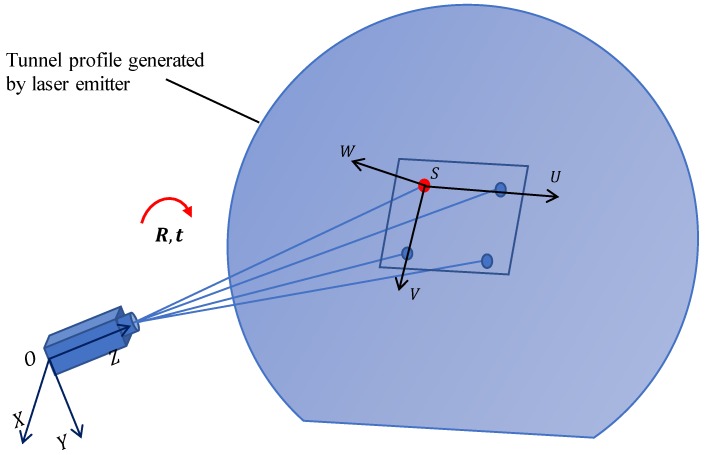
Calibration of relationship between the camera and the tunnel profile.

To calibrate the relationships of all cameras and the tunnel profile, we only need to calibrate the co-ordinate system between one camera and the tunnel profile, as we have calibrated the transformation matrix between the cameras in process (2). A square target coincident with the tunnel profile is used in this calibration. The square target establishes a target co-ordinate system (TCS) SUVW, which is equal to the co-ordinate system of the tunnel profile $S_{p}$. Thus, there should be a transformation matrix R,t between TCS SUVW and CCS OXYZ, as shown in Figure 9. As the geometric information of the square target and the camera parameters are known, the transformation matrix R,t can be computed. Then, the tunnel profile $S_{p}$ in CCS OXYZ can be expressed by:

(11) $$u_{xz}\cdot x+u_{yz}\cdot y+u_{zz}\cdot z=u_{xz}\cdot t_{x}+u_{yz}\cdot t_{y}+u_{zz}\cdot t_{z}.$$

## 4. On-Site Application

### 4.1. Introduction of On-Site Application

For this study, on-site applications were conducted from December 2012 to June 2013. Four identical non-metric cameras, each with resolution of 1600×1200 (15 fps), were equipped on the integrated device. The device was set to acquire one cross-sectional deformation per meter and the whole inner surface image of the tunnel.

The proposed device and methods were validated in several tunnels of Shanghai Metro. These metro tunnels were all constructed by the shield tunneling method and they are all buried in soft soil. These tunnels are circular tunnels, assembled by segmental linings with a inner diameter of 5500 mm. Due to construction activities beside the metro tunnel, these tunnels have observed severe surface defects and cross-sectional deformations. Manual inspection was also conducted at the same time, for comparison with the rapid inspection method.

**Figure 10:**
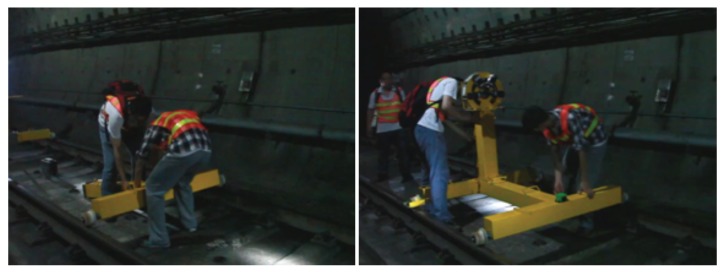
Assembling the device during the on-site application.

As mentioned above, the integrated device was systematically designed for rapid assembly and disassembly, which took about 20 min in total. Figure 10 shows the quick assembly of the integrated device during the on-site application.

The function of acquiring two kinds of structural defects was validated during the on-site application. The device was able to simultaneously acquire images of the tunnel profile generated by laser emitter and images of surface defects under illumination, as shown in Figure 2.

The speed of the integrated device was 0.2 h/km (5 km/h), in the case of acquiring the surface defects of the metro tunnel and 1000 cross-sectional profiles. For comparison, there is a typical time cost of 1.5 h/km for graphing the surface defects of a metro tunnel by manual inspection, while measuring 10 cross-sectional profiles by total station. It is obvious that the speed of proposed device was much faster than the traditional manual method.

### 4.2. Results of Identified Surface Defects

**Figure 11:**
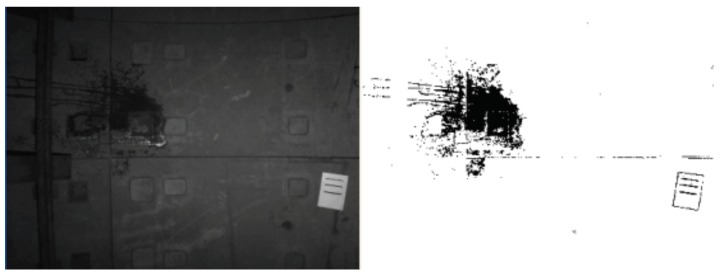
Extraction of man-made leakage in the metro tunnel.

Firstly, the image differencing strategy was validated by a man-made leakage in the metro tunnel, as shown in Figure 11. The result shows that image differencing strategy could accurately extract changes on the surface of the metro tunnel.

**Figure 12:**
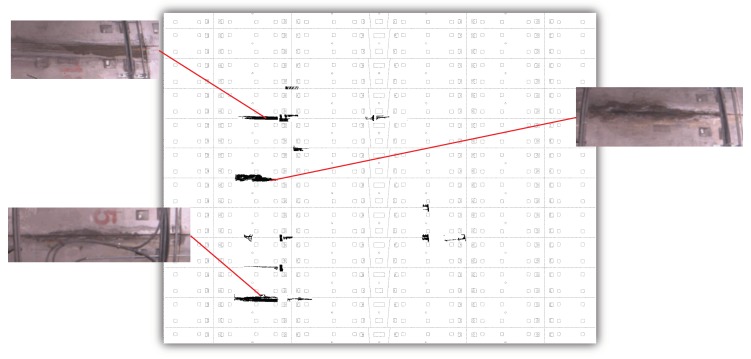
Identification and validation of real leakages in the metro tunnel.

To intuitively display the identified surface defects, an expansion map of the inner surface of the metro tunnel was drawn proportionally, as shown in Figure 12. The images of real leakages in the metro tunnel were geometrically compared with the identified results, which showed very good consistency in shapes and edges. However, mainly due to the insufficient resolution of the non-metric camera, cracks and spallings in the metro tunnel were not successfully extracted during the on-site application.

**Figure 13:**
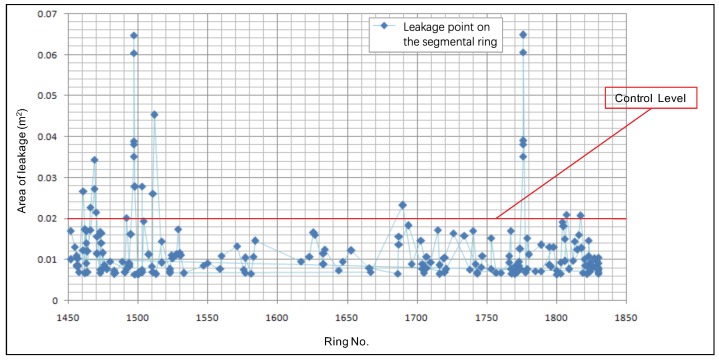
Areas of identified leakages in the metro tunnel.

As there is a proportional relationship between pixels and square meters (m${}^{2}$), the areas of identified leakages in Shanghai Metro Line 7 are plotted in Figure 13.

Monitoring measures or repairing measures should be taken on large individual leakages detected by inspection. Setting an area of 0.02 m${}^{2}$ as the control level for leakages, there were 23 critical leakages identified during the on-site application. After a comprehensive analysis after inspection, these leakages were concurrent with large deformations, mainly caused by construction activities adjacent to the metro tunnel.

### 4.3. Results of Identified Cross-Sectional Deformation

The results of identified cross-sectional deformations by the proposed method are validated by measurement results of the total station in several aspects.

**Figure 14:**
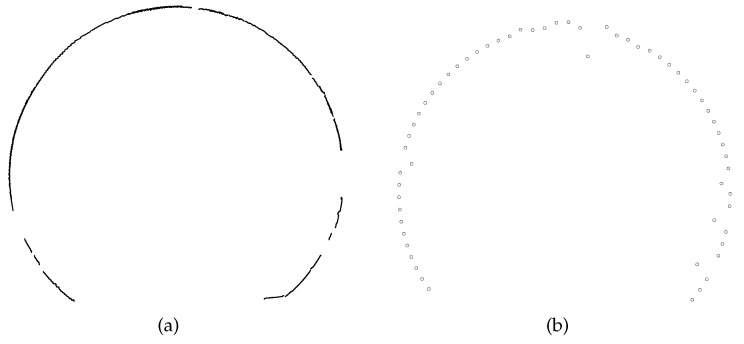
Identified cross-sectional profile of metro tunnel: (**a**) By the proposed method and (**b**) by the total station.

Complete acquisition of the cross-sectional profile is the basis of computing the geometric information. There were about 3000 co-ordinates acquired and computed by the proposed method. For comparison, there were about 50 measured points by the total station. The cross-sectional profiles identified by the two methods are presented in Figure 14. It can be seen that the curve of the tunnel profile identified by proposed method is more continuous and both of the two profiles contain noises and discontinuous parts. These noises and noncontinuous parts are mainly caused by power, ventilation and signal lines installed within the metro tunnel.

**Figure 15:**
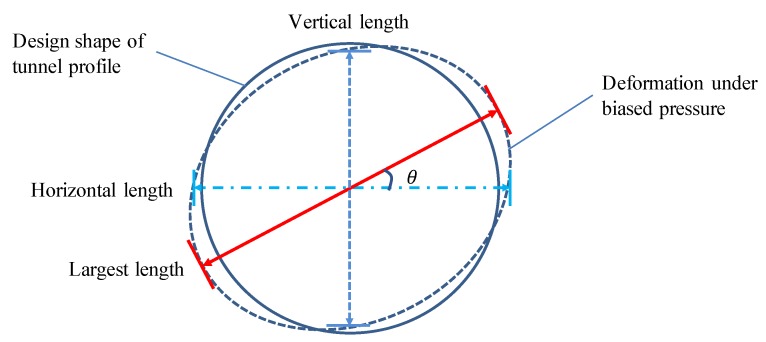
The assumed cross-sectional profile of a deformed metro tunnel.

Geometric information of the cross-sectional profile should be known, in order to assess the state of the tunnel’s structure. In practice, the cross-sectional deformation (or convergence) is usually adopted as an indicator to assess the state of the tunnel structure. As the cross-sectional profile of a deformed metro tunnel is usually assumed to be an ellipse, as shown in Figure 15, a geometric fitting method is proposed.

The co-ordinates of the pixels are fitted by Equation (12), which describes a general ellipse.

(12) $$\left\{\begin{array}{c} \left[\begin{array}{c} x-x_{0}\\ y-y_{0} \end{array}\right]=\left[\begin{array}{cc} \cos\theta & \sin\theta \\ -\sin\theta & \cos\theta \end{array}\right]\left[\begin{array}{cc} a & 0\\ 0 & b \end{array}\right]\left[\begin{array}{c} x_{e}\\ y_{e} \end{array}\right]\\ x_{e}^{2}+y_{e}^{2}=1 \end{array}\right.,$$

where $\left(x_{0},y_{0}\right)$ is the center of the ellipse, *a* is major-axis length of the ellipse, *b* is minor-axis length of the ellipse and θ is the angle between the major-axis of the ellipse and X-axis of the co-ordinate system. The parameters of the ellipse can be estimated by the least-squares method. Thus, the cross-sectional deformation can be assessed by the difference of the fitted ellipse and the designed shape of the metro tunnel.

**Figure 16:**
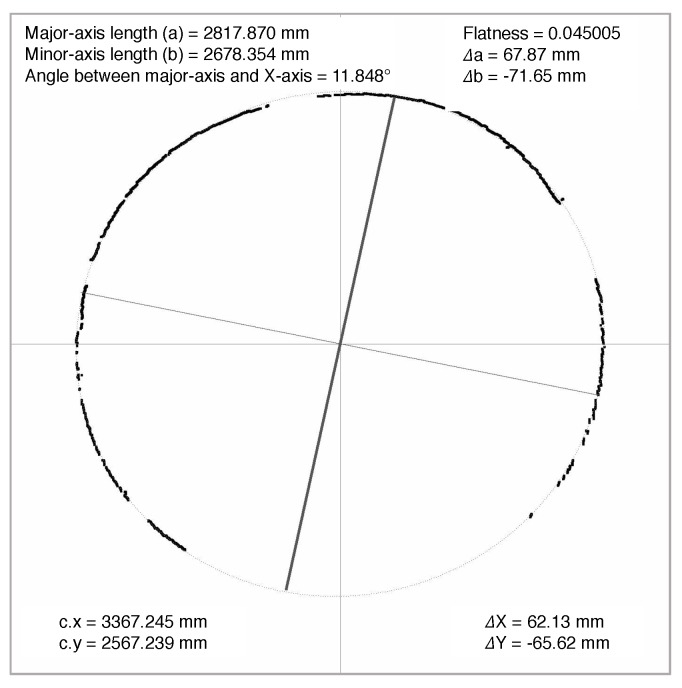
Identified cross-sectional deformation in metro tunnel.

As shown in Figure 16, the geometric information of fitted ellipse is intuitively printed on the image. The major-axis length of the ellipse is 2817.870 mm, the minor-axis length of ellipse is 2678.354 mm and the angle between major-axis of the ellipse and the X-axis of the co-ordinate system is 11.848. The cross-sectional deformation Δ was calculated by Equation (13), where *r* is the designed radius of the circular metro tunnel:

(13) Δ=2×(a−r).

**Figure 17:**
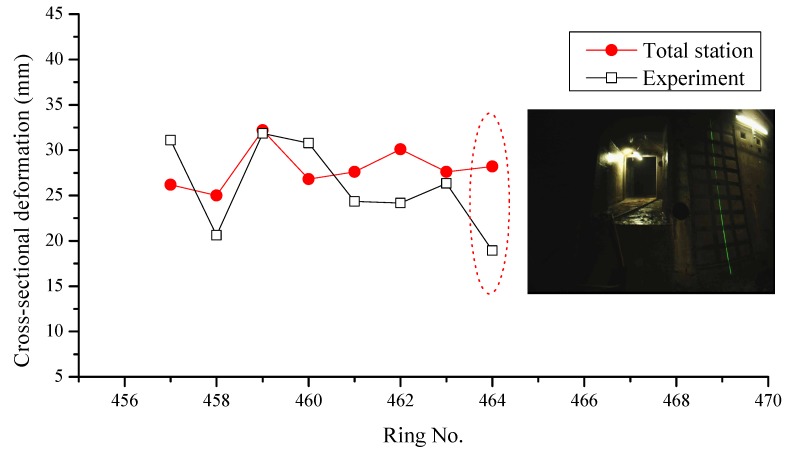
Discrepancy of cross-sectional deformation close to a cross-passage of the metro tunnel.

The co-ordinates of the points measured by the total station were also processed by geometric fitting. Based on the on-site application in Shanghai Metro Line 12, the results of several cross-sectional deformations, as calculated by the two methods, were compared and analyzed. Cross-sectional deformation at eight positions was identified and compared, as shown in Figure 17. The discrepancies of the proposed method and the total station were irregular, with a mean value of 1.97 mm and standard deviation of 4.81 mm.

The discrepancies of the proposed method and the total station were caused by several reasons. Firstly, the cross-sectional profile generated by integrated device was vertical to the axis of the rail-track but the profile measured by total station was coincident with the direction of gravity. Thus, the profiles measured by the two methods were different. Secondly, the occurrence of a cross-passage interfered with the acquisition of profile images and introduced noise into the calculation of the geometric information. As shown in Figure 17, the discrepancy close to a cross-passage of the metro tunnel was larger than those at other positions.

**Figure 18:**
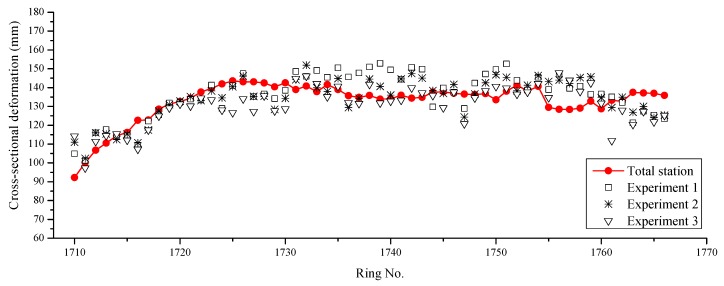
Comparison between the proposed method and the total station.

Three individual experiments were conducted in a particular section to investigate the accuracy of proposed method in Shanghai Metro Line 7. Figure 18 shows the results of the total station and three experiments using the proposed method. It can be seen that the discrepancies between them were not large. The statistics of the discrepancies between the experiments and the total station are listed in Table 2. The standard deviations of the discrepancies were consistent, indicating that the proposed method is stable. Compared with the results for Shanghai Metro Line 12, the discrepancies of the results for Shanghai Metro Line 7 were larger, mainly due to the increment of data volume.

Generally, the results measured by the total station were closer to the real values. Thus, the accuracy of the proposed method can also be indicated from the results of the on-site application in Shanghai Metro Line 7, which had a standard deviation of 8.97 mm for a single measurement. Based on the Pauta criterion (3σ criterion), this standard deviation of the discrepancy means that the proposed method can identify cross-sectional deformation larger than 27 mm within a 99.73% confidence interval.

According to the structural appraisal standards of a shield tunnel [52], the cross-sectional deformation is an important indicator for assessment of the service state of a shield tunnel. Cross-sectional deformation larger than 10‰·D can be defined as a “severe structural defect,” for which repair measures need to be taken, as listed in Table 3. As the diameter *D* of the shield tunnel in the on-site application was 5500 mm, severe structural defects was calculated as deformation larger than 55 mm. Thus, the proposed method is able to screen out severe cross-sectional deformations of the metro tunnel with acceptable accuracy.

### 4.4. Comparison with the State-of-the-Art Works

In current situation, the methods based on optimal imaging and laser scanning become more and more popular [23,34,35,37,38,44,45,46]. Additionally, some advanced techniques, such as Deep Learning, were applied to identify the surface defects in tunnel [36]. Several state-of-the-art works are listed in Table 4. It can be seen that the proposed method is able to identify two kinds of defects, however, other methods are only specific to one kind of defects.

The performance of proposed method and the aforementioned state-of-the-art works are compared in Table 5. Some results are: (1) The application of fully convolutional network (FCN) greatly improves the accuracy of identifying surface defects, such as leakage, which is a promising research direction. (2) The performance of laser scanning method (M-4, M-5 or M-6) highly depends on the device. The technical solution using a Terrestrial Laser Scanning (TLS) is much better than Light Detection and Ranging (LiDAR) but the speed of LiDAR can be very fast. (3) The accuracy of propped method achieved an acceptable level.

After all, it can be concluded that the proposed method has comparative advantages than other methods in the aspect of multi-functional, rapid speed and acceptable accuracy.

### 4.5. Analysis of Cost and Efficiency

From the results of on-site application, the cost, accuracy and efficiency of proposed method can be compared with manual inspection (for surface defects) and total station measurement (for cross-sectional deformation), as listed in Table 6.

Comprehensively considering the indicators of labor cost and accuracy, it is obvious that the proposed method is superior to the traditional methods. Regarding surface defects, manual inspection required more labor costs, both on-site and in office and the results seriously depend on the professionalism of the inspector. In contrast, the proposed method can reduce human error effectively, at high speed. Regarding the cross-sectional deformation, the proposed method has much lower labor costs than the total station method. Even though the accuracy of proposed method was not better than the total station, it is accurate enough for screening out serious cross-sectional deformations in metro tunnels. Thus, this method will greatly improve the efficiency of inspection of metro tunnels.

## 5. Conclusions

This study proposed an integrated device for rapidly acquiring and identifying two kinds of structural defects (surface defects and cross-sectional deformation) in metro tunnels. The conclusions are drawn, as follows:

- With a systematic consideration of integrity and rapidity, the design of the integrated device achieved the functional requirements, which were rapid and simultaneous acquisition of two kinds of structural defects during the moving process.
- The identification method, based on image differencing strategy, was able to rapidly extract leakages in the metro tunnel. However, the cracks and spallings were not able to be extracted, mainly due to the insufficient resolution of the cameras.
- The identification method, based on transmissive projection, was able to rapidly measure cross-sectional deformations of metro tunnel.
- The device and proposed method can greatly reduce labor costs, which improves the efficiency of inspection.

In the future, some improvements on this study, such as upgrading the resolution of the cameras, optimization of algorithms and vibration control of the integrated device, are worth doing in order to expand the application of the integrated device and proposed method.

## Figures and Tables

**Table 1:** Features of different types of defects.

Type of defect	Leakage	Spalling	Crack
Area	Large	Medium	Small
Length of long axis	Large	Medium	Large
Length of short axis	Medium	Medium	Small
Fill rate	Large	Large	Small
Long axis/Short axis	Large	Small	Large

**Table 2:** Statistics of the discrepancies of the proposed method.

Statistics	Experiment 1	Experiment 2	Experiment 3
Mean	−3.21	−1.59	2.35
Standard deviation	8.97	8.10	8.94

**Table 3:** Assessment of the service state of the shield tunnel.

Ranges of Cross-Sectional Deformation	Service State	Corresponding Measures
<10‰·D	i,ii	No repair
10∼15‰·D	iii	Minor repair
15∼25‰·D	iv	Medium repair
>25‰·D	v	Major repair

**Table 4:** Methodologies of state-of-the-art works.

Methods	Sensors	Algorithms	Identified Defects	Reference
Proposed	CCD Camera	(1) Image differencing; (2) Transmissive projection	(1) Leakage; (2) Deformation	N.A.
M-1	CMOS Camera	Morphological image processing	Crack	[35]
M-2	CMOS Camera	Gabor filter invariant to rotation	Crack	[34]
M-3	CMOS Camera	Fully convolutional network (FCN)	Leakage	[36]
M-4	Laser scanner	Mesh modeling algorithm	Deformation	[23]
M-5	Laser scanner	Extraction of cross-sectional	Deformation	[37,44,45]
M-6	Laser scanner	Least squares adjustment	Deformation	[38,46]

**Table 5:** Comparison of proposed method with state-of-the-art works.

Methods	Technical Solution	Speed	Accuracy
Proposed	Removable cart equipped with cameras	>5 km/h	(1) Error rate ≈ 10%; (2) RMSE ≈ 9 mm
M-1	Train carriage equipped with cameras	N.A.	Error rate < 10%
M-2	Removable cart equipped with cameras	<0.9 m/s	Error rate < 5%
M-3	Removable cart equipped with cameras	0–10 km/h	Error rate < 2%
M-4	Terrestrial Laser Scanning (TLS) -static mode	N.A.	Close to total station
M-5	Terrestrial Laser Scanning (TLS) -kinematic mode	Walking speed	RMSE: 0.8–4.8 mm
M-6	Train carriage equipped with Light Detection and Ranging (LiDAR)	120 km/h	RMSE: 0.02–0.03 m

**Table 6:** Comparison of inspection methods for a metro tunnel (per kilometer).

Indicators	Manual Inspection	Total Station Method	Proposed Method
Labor cost (on-site)	1.5 h	30 h	0.2 h
Labor cost (in office)	4.0 h	0.1 h	0.1 h
Accuracy	Depend on manual qualification	2 to 5 mm	about 27 mm
